# How to Perform and Prepare for Robotic Sleeve Lobectomy

**DOI:** 10.1016/j.atssr.2023.03.015

**Published:** 2023-04-03

**Authors:** Kohei Hashimoto, Junji Ichinose, Yosuke Matsuura, Masayuki Nakao, Sakae Okumura, Mingyon Mun

**Affiliations:** 1 Department of Thoracic Surgical Oncology, Cancer Institute Hospital, Japanese Foundation for Cancer Research, Tokyo, Japan

## Abstract

Compared with video-assisted thoracic surgery, robotic surgery may be particularly useful for reconstructive procedures such as sleeve lobectomy. However, specific training for robot-specific tricks and pitfalls is warranted. Using running suture with a short double-armed suture, we have demonstrated the feasibility of simulating robotic sleeve resection with a previously developed 3-dimensional operable airway model. This training can be done in any institution with robots with flexible timing for the busy thoracic surgeon. The durability and synthetic nature of this model allow repeated training. This can help surgeons to be well prepared for robotic sleeve lobectomy.

The feasibility of bronchoplasty by video-assisted thoracic surgery or a robotic approach has been reported.^1^, ^2^, ^3^, ^4^ Theoretically, robot-assisted surgery would be particularly useful for reconstructive procedures because of its maneuverability and stereoscopic vision. However, as surgical robots have their own benefits and pitfalls, specialized training is warranted. The animal laboratory is a popular method, but this can be done only in specialized facilities. There is also the associated cost and time for travel, which limits the opportunity. We have previously reported on 3-dimensional (3D) airway models created with human computed tomography data that can replicate open sleeve resection and anastomosis procedures.^5^ In our model, the surgical feel was shown to be reasonable as assessed by multiple board-certified thoracic surgeons. This system can also be customized with patient-specific anatomy for the purpose of “patient-specific preoperative simulation.”^6^ In this report, the simulation system was applied to robotic sleeve resection.

## Technique

The creation of an airway model has been described previously.^5^ Briefly, this was designed in 3D from non–contrast-enhanced computed tomography images of a healthy male volunteer. Plastic models were 3D printed, and silicone molds were created on the basis of the plastic models. Urethane materials mimicking the cartilage and remaining connective tissue (including the membranous portion) were poured into the molds while the 2 parts were combined. This study was approved by the institutional review board on May 28, 2021, and informed consent was waived.

**Figure 1:**
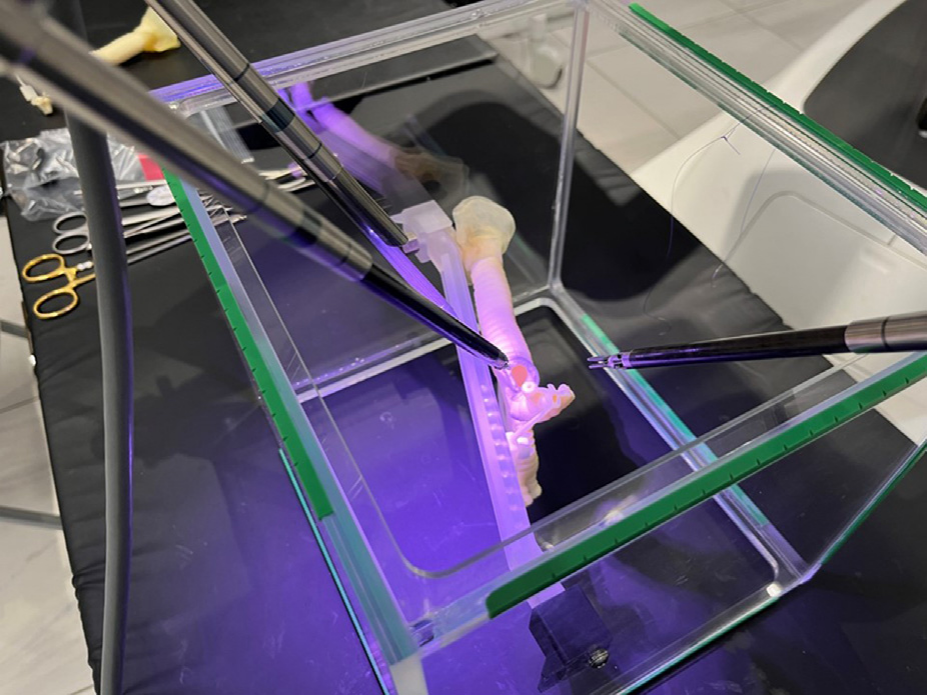
Simulation of robotic sleeve lobectomy using a 3-dimensional airway model.

**Figure 2:**
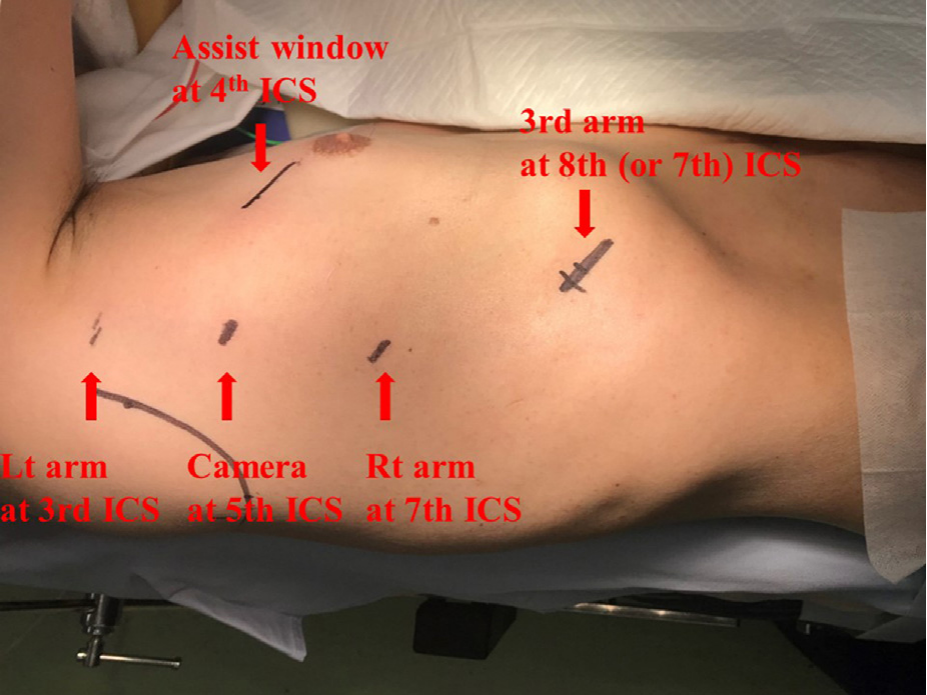
Our port placement for the right-sided robotic lobectomy. A camera port is placed at the fifth intercostal space (ICS) on the posterior axillary line to better visualize the hilar anatomy. (Lt, left; Rt, right.)

**Figure 3:**
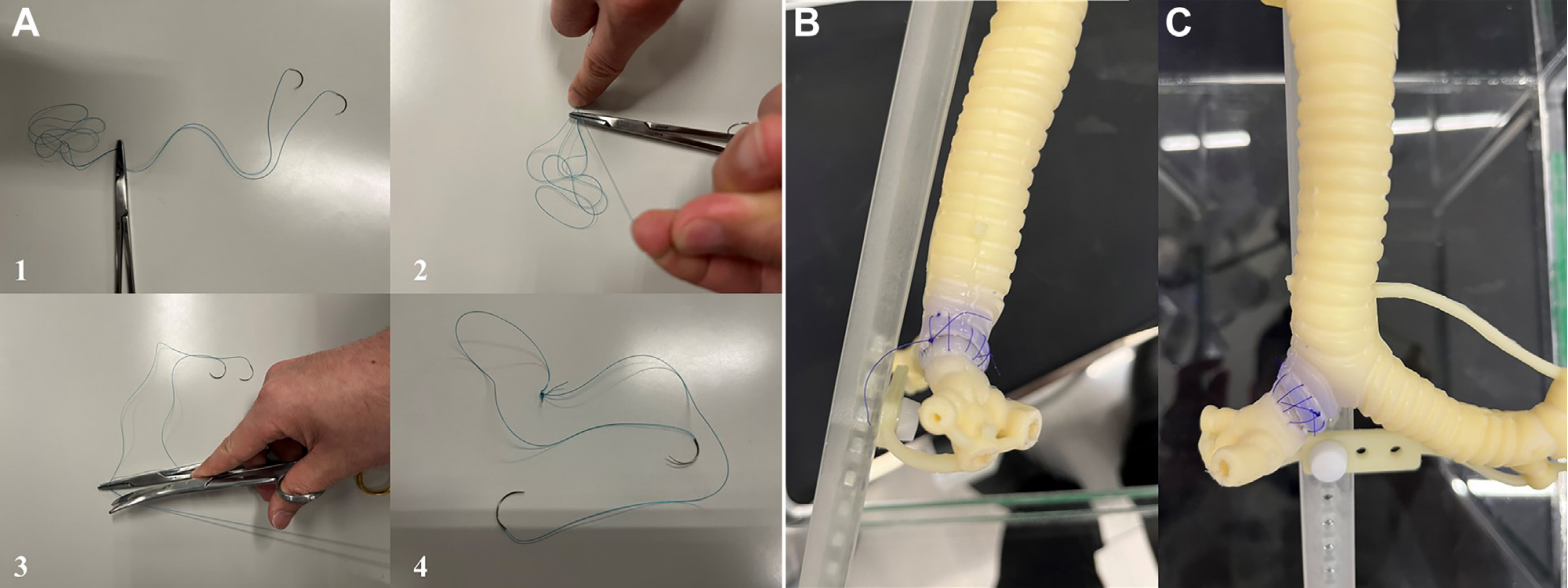
(A) 1. Using a needle holder, grasp double-armed sutures 15 cm from the needles. 2. Tie the two arms together 6 times to form a tight knot. 3. Cut the excess suture on the other side of the needle holder. 4. Complete the making of short double-armed suture. (B) Anastomosed 3-dimensional airway model using a 4-0 monofilament suture, right-side view. (C) Anastomosed 3-dimensional airway model using a 4-0 monofilament suture, front view.

The da Vinci Xi (Intuitive Surgical) surgical system was used with the robotic ports fixed in the air (Figure 1). This configuration was set similar to our clinical port placement for right-sided robotic lobectomy (Figure 2). The procedure of right upper sleeve resection and anastomosis was performed by a board-certified thoracic surgeon (Video). The anastomosis was performed by continuous suturing with use of a DeBakey forceps in the left hand and a large needle driver in the right hand. Another retraction arm (third arm) was used with a Cadiere forceps. A short (approximately 25 to 30 cm) double-armed 3-0 woven (alternatively 4-0 monofilament) suture was used for the anastomosis. This short double-armed suture was created by tying 2 suture tails 15 cm from the arm (Figure 3A). First, the needle was placed “out-in” at the proximal cut end at the ventral side of the cartilaginous portion until the knot connecting 2 short sutures was suspended on the cartilage. This knot served as a stay while the running suture was performed in the next step. The mediastinal side of the cartilaginous portion through the membranous portion to the lateral portion of the cartilaginous portion was continuously anastomosed. Next, the other side of the suture arm was used for continuous anastomosis of the remaining cartilaginous portion. Care was taken to take 1 cartilage ring width as a bite. The sutures were tightened with a hook-shaped monopolar cautery (like a nerve hook) before tying a knot. A knot was tied outside the bronchus using robotic instruments. There was no cutting of the sutures or the model (Figures 3B, 3C). This was also replicated with both woven and monofilament sutures.

## Comment

In this technical report, we have demonstrated the feasibility of simulation for robotic sleeve resection using a 3D operable airway model that we have previously developed. We tried different anastomosis techniques, such as interrupted sutures, running sutures, or a combined technique. We found that running sutures were suitable for robotic bronchial anastomosis because it was difficult to organize many sutures during interrupted sutures with robotic arms. Also, the standard thread of 75- to 90-cm-long suture was difficult to follow in the surgical field during anastomosis. Reports in a lung cancer field^1^^,^^3^^,^^4^^,^^7^ as well as in a lung transplant field^8^ showed the safety of bronchial anastomosis using running sutures; this running suture does not seem to be a problem in the short or long term.

There are multiple advantages of using the 3D airway model that we have developed over existing laboratory training methods. First, it can be done at each hospital with robots and with flexible timing. This should help busy thoracic surgeons and trainees. Also, airways taken from animals are considered a biohazard; as such, using a robot intended for clinical use on such a substrate is not ideal. Second, the anatomy is also substantially different between humans and other animals. A special holder that replicates the surgical exposure of the airway also makes a difference during training. Third, the models can be used repeatedly because of durability of the model for manipulation and its synthetic nature (the model has not deteriorated for at least 1 year since we developed it). The anastomosis can be performed approximately 10 times per each cut surface. Further training is possible by cutting a cartilage ring and creating a fresh surface. As training requires repetition, this is particularly important for trainees. We are now in the process of releasing this training system as a commercial product for both domestic (in Japan) and international use. At the development stage, this 3D airway model costs approximately USD 500 (CrossEffect, Kyoto, Japan). Efforts will be made to reduce the cost at the distributing phase to maximize the cost-effectiveness of this training method. Because various types of tracheobronchial resection and reconstruction have been performed on the models during an open procedure, universal tracheobronchial reconstruction training should also be possible with the robots.

In conclusion, this simulation system with a 3D operable airway model may be useful for robotic bronchoplasty.
